# Inhibition of CD26/DPP-IV enhances donor muscle cell engraftment and stimulates sustained donor cell proliferation

**DOI:** 10.1186/2044-5040-2-4

**Published:** 2012-02-16

**Authors:** Maura H Parker, Carol Loretz, Ashlee E Tyler, Lauren Snider, Rainer Storb, Stephen J Tapscott

**Affiliations:** 1 Program in Transplantation Biology, Clinical Research Division, Fred Hutchinson Cancer Research Center, 1100 Fairview Avenue N, Mailstop D1-100, Seattle, WA, 98109-1024, USA; 2 Human Biology Division, Fred Hutchinson Cancer Research Center, 1100 Fairview Avenue N, Mailstop C3-168, Seattle, WA, 98109-1024, USA; 3 Department of Medicine, Health Sciences Building RR-512, University of Washington, 1959 NE Pacific Street, Box 356420, Seattle, WA, 98195, USA; 4 Department of Neurology, Health Sciences Building RR-650, University of Washington, 1959 NE Pacific Street, Box 356465, Seattle, WA, 98195, USA

**Keywords:** muscular dystrophy, cell transplantation, xenotransplant, canine, CXCR4, diprotin A

## Abstract

**Background:**

Transplantation of myogenic stem cells possesses great potential for long-term repair of dystrophic muscle. In murine-to-murine transplantation experiments, CXCR4 expression marks a population of adult murine satellite cells with robust engraftment potential in *mdx *mice, and CXCR4-positive murine muscle-derived SP cells home more effectively to dystrophic muscle after intra-arterial delivery in *mdx^5cv ^*mice. Together, these data suggest that CXCR4 plays an important role in donor cell engraftment. Therefore, we sought to translate these results to a clinically relevant canine-to-canine allogeneic transplant model for Duchenne muscular dystrophy (DMD) and determine if CXCR4 is important for donor cell engraftment.

**Methods:**

In this study, we used a canine-to-murine xenotransplantation model to quantitatively compare canine muscle cell engraftment, and test the most effective cell population and modulating factor in a canine model of DMD using allogeneic transplantation experiments.

**Results:**

We show that CXCR4 expressing cells are important for donor muscle cell engraftment, yet FACS sorted CXCR4-positive cells display decreased engraftment efficiency. However, diprotin A, a positive modulator of CXCR4-SDF-1 binding, significantly enhanced engraftment and stimulated sustained proliferation of donor cells *in vivo*. Furthermore, the canine-to-murine xenotransplantation model accurately predicted results in canine-to-canine muscle cell transplantation.

**Conclusions:**

Therefore, these results establish the efficacy of diprotin A in stimulating muscle cell engraftment, and highlight the pre-clinical utility of a xenotransplantation model in assessing the relative efficacy of muscle stem cell populations.

## Background

Duchenne muscular dystrophy (DMD), the most common and severe form of muscular dystrophy, is caused by mutations in the dystrophin gene, the largest gene identified in the human genome. Transplantation of myogenic stem cells possesses great potential for long-term repair of dystrophic muscle. Indeed, intramuscular injection of adult satellite cell-derived myoblasts from a normal syngeneic donor into *mdx *mice results in the formation of dystrophin-positive muscle fibers [1-3]. However, in small-scale human clinical trials, intramuscular injection of donor myoblasts resulted in transient expression of dystrophin in a small number of recipient muscle fibers and triggered cellular immune responses that destroyed newly-formed donor myotubes [4-8].

We used a clinically acceptable regimen of hematopoietic stem cell transplantation to establish mixed donor/host blood cell chimerism and immune tolerance in a canine model of Duchenne muscular dystrophy (*cxmd*) [9]. Intramuscular injection of donor muscle-derived cells into chimeric *cxmd *recipients restored dystrophin expression for at least 24 weeks in the absence of post-transplant immunosuppression, indicating that cell transplantation may be a viable therapeutic option for muscular dystrophy. Yet, it remains unclear from murine transplantation experiments which cell population most effectively engrafts into diseased skeletal muscle.

Embryonic myogenic progenitor cells express CXCR4, G-protein coupled cell surface receptor, and migrate towards regions of SDF-1 expression during limb muscle development, suggesting that CXCR4/SDF-1 plays a role in muscle cell homing. Indeed, CXCR4-positive muscle-derived side population (SP) cells home more effectively to dystrophic muscle after intra-arterial delivery in *mdx^5cv ^*mice [10]. Moreover, CXCR4 expression marks a population of adult satellite cells with robust engraftment potential in *mdx *mice [11]. Together, these data suggest that CXCR4 plays an important role in donor cell engraftment.

We used the xenotransplant model to show that CXCR4 expression on canine donor muscle cells is also important for cell engraftment, but FACS sorting for CXCR4-positive cells decreased their engraftment efficiency. Diprotin A stimulation of CXCR4, however, significantly increased the number of canine dystrophin-positive muscle fibers and canine-derived satellite cells by enhancing donor cell proliferation. Moreover, xenotransplantation accurately predicted results in canine-to-canine allogeneic transplantation experiments, an important pre-clinical model for future human clinical trials.

## Methods

### Canine donor cell isolation

The Institutional Animal Care and Use Committee at the Fred Hutchinson Cancer Research Center, which is fully accredited by the Association for Assessment and Accreditation of Laboratory Animal Care, approved this study. The biceps femoris muscle of a wild-type canine was biopsied as a survival surgery, and the biopsy was first digested with 200 U/ml collagenase type 4 (Worthington Biochemical, Lakewood, NJ, USA) in Dulbecco's Modified Eagle Medium (DMEM; Invitrogen, Carlsbad, CA, USA) supplemented with 5 mM CaCl_2_, 1 U/ml dispase (Invitrogen), and 0.5% BSA for 30 min at 37°C. The intact fibers and muscle pieces were rinsed in Hank's Balanced Salt Solution (HBSS; Invitrogen) and transferred to 400 U/ml collagenase type I (Sigma-Aldrich, St. Louis, MO, USA) in DMEM (Invitrogen) supplemented with 5 mM CaCl_2 _for 45 min at 37°C. The digested muscle was triturated and filtered through a series of nylon mesh filters, and the resulting mononuclear cells washed twice in phosphate buffered saline (PBS), and resuspended in PBS.

### FACS

Anti-CXCR4 was obtained from R & D Systems (clone 44716; Minneapolis, MN, USA) and used at 10 μg/ml for FACS sorting of 4-6 × 10^6 ^cells. PE-labeled anti-mouse IgG2b was obtained from SouthernBiotech (Birmingham, AL, USA) and used at 0.25 μg/ml. Freshly isolated canine skeletal muscle cells were resuspended in FACS buffer (Hanks Balanced Salt Solution [HBSS], 5% FBS) and incubated on ice with anti-CXCR4 or isotype control, followed by PE-labeled anti-mouse IgG. The cells were washed and resuspended in FACS buffer containing 1 μl of 5 mM SYTOX^® ^Blue (Invitrogen). The cells were sorted using a BD Aria II, and the population of CXCR4-positive:SYTOX blue-negative cells were collected and prepared for injection.

### Cell injection into mice and tissue processing

The right hindlimb of each 7 to 12-week-old NOD/SCID mouse was exposed to 12 Gy of ionizing irradiation (Mark 1 cesium source, Sheppard and Associates, San Francisco, CA, USA), and the tibialis anterior (TA) muscle of the same hindlimb was injected with 50 μl of 1.2% barium chloride immediately after irradiation. The following day, the same TA muscle was injected with 50 μl of canine cells along the length of the muscle, so as to distribute cells from the distal to the proximal end of the muscle. Where noted, mice were administered 100 μg EdU intraperitoneally daily for 7 days. Injected muscle was harvested 28 days after injection unless otherwise indicated. Harvested mouse muscle was covered in OCT within a plastic cryomold and placed on top of an aluminum block immersed in liquid nitrogen. Cryosections were cut (8 to 10 μm) from the distal to the proximal end of the frozen muscle using a Leica CM1850 cryostat, and adhered to Superfrost slides (Fisher Scientific, Pittsburgh, PA, USA). Each glass slide consisted of four serial sections, and the corresponding section on the subsequent slide represented a separation of approximately 200 μm from the previous slide.

Each TA muscle normally generated 24 slides, each consisting of four serial sections. Initially, slides 6, 12, and 18 were stained for dystrophin and lamin A/C to determine the region of highest engraftment. Three more even-numbered slides were chosen from the region of highest engraftment and stained for canine dystrophin and lamin A/C. Three odd-numbered slides in the same region were used for Pax7 and lamin A/C co-staining. In almost all cases, the region of highest engraftment was between slides 6 and 18, representing the belly of the muscle, which does not vary considerably in cross-sectional area.

### Immunostaining

Primary antibodies specific for dystrophin were used at a 1:50 dilution, and included MANDYS107 (4H8), and MANEX1A (4C7), developed by Glenn Morris and obtained from the Developmental Studies Hybridoma Bank developed under the auspices of the NICHD and maintained by The University of Iowa, Department of Biological Sciences, Iowa City, IA, USA. Anti-lamin A/C (clone 636) was used at 1:100 and was obtained from Vector Laboratories (Burlingame, CA, USA). Alexa fluor 488-conjugated goat anti-mouse IgG and Alexa fluor 568-conjugated goat anti-mouse IgG2b secondary antibodies, both from Invitrogen, were used at 1:200. For dystrophin and lamin A/C staining, the sections were fixed in acetone at -20°C for 10 min, allowed to dry, and rehydrated in PBS. Sections were blocked in 1× PBS buffer containing 2% goat serum, 1% BSA, 0.1% cold fish skin gelatin, and 0.05% sodium azide, followed by incubation with primary antibody diluted in 1× PBS containing 1% BSA, 0.1% cold fish skin gelatin, and 0.05% sodium azide. Where appropriate, EdU was visualized using the Click-iT EdU Alexa Fluor 647 Imaging kit according to the manufacturer's instructions (Invitrogen). The sections were incubated with secondary antibody diluted in 1× PBS and mounted with ProLong Gold Anti-fade with DAPI (Invitrogen).

Primary antibody specific for Pax7 antibody was used at 1:10, and was obtained from the Developmental Studies Hybridoma Bank. Rabbit polyclonal antibody to laminin was obtained from Abcam and used at 1:100. Alexa fluor-conjuagted goat anti-mouse IgG1 (Pax7), Alexa fluor 568-conjugated goat anti-mouse IgG2b (lamin A/C), and Marina blue-conjugated goat anti-rabbit (laminin) were used at 1:200, and obtained from Invitrogen. For Pax7, laminin, and lamin A/C co-staining, cryosections were fixed in 4% paraformaldehyde for 20 min at room temperature, washed with 1× PBS, followed by permeabilization with methanol at -20°C for 6 min. The sections were washed in 1× PBS, and antigen retrieval was performed by incubating the slides twice in 10 mM citric acid (pH 6.0) at 90°C for 5 min. Sections were blocked and stained as described above.

Photomicrographs were taken using either a Nikon E800 and a CoolSnap HQ camera, or a Zeiss AxioImager.Z1 as part of a TissueFaxs system (TissueGnostics, Los Angeles, CA, USA). The images for each field of view were stitched together to form an entire cross-sectional view. Counts of fibers expressing canine dystrophin, nuclei expressing canine lamin A/C, and nuclei co-expressing Pax7 and canine lamin A/C were done manually, and curve fitting was performed using Microsoft Excel.

### Primary myoblast culture and immunocytochemistry

Canine muscle and mouse muscle injected with canine cells were chopped in 400 U/ml collagenase type I (Sigma-Aldrich, St Louis, MO, USA) in DMEM and incubated at 37°C for 45 min. The mixture was triturated and filtered using a series of nylon mesh filters. The resulting cells were cultured in growth medium (Ham's F10, 20% FBS, penicillin/streptomycin, 2.5 ng/ml bFGF). Once confluence was reached, cultured cells were fixed in 4% paraformaldehyde, and permeabilized with 0.3% Triton X-100 in 1× PBS. Primary antibodies specific for Pax7 and myogenin (F5D) were obtained from the Developmental Studies Hybridoma Bank. Cells were blocked in 10% goat serum, and incubated with primary antibody diluted 1× PBS containing 1% BSA, 0.1% cold fish skin gelatin, 0.05% sodium azide, 1× PBS. The cells were washed in 1× PBS, incubated with secondary antibody and mounted with ProLong Gold Anti-fade with DAPI (Invitrogen). Photomicrographs were taken using a Nikon E800 and CoolSnap HQ camera.

### RNA isolation and RT-PCR

RNA was isolated using the RNeasy Kit (Qiagen, Valencia, CA, USA) and 250 ng reverse transcribed using SuperScript III (Invitrogen) and random primers. PCR was performed using PlatinumTaq (Invitrogen) and one-tenth of the reaction mix for 30 cycles with the following primers: MyoD F1-CGATTCGCTACATCGAAGGT, MyoD R1-AGGTGCCATCGTAGCAGTTC; CD26/DPP IV F1-GTGTCTCCCGATGGACAGTT, CD26/DPP IV R1-CCCGTTCCATGTGATTCTCT; SDF-1 F1-CAGCCTGAGCTACAGATGTCC, SDF-1 R1-CTTCAATTTCGGGTCAATGC; CXCR4 F1-GAGCTCCATATATACCCT TCAGAT A, CXCR4 R1-GGTAACCCATGACCAGGATG; Pax7 F1-AAGATTCTCTGCCGCTAC CA, Pax7 R1-TCACAGTGTCCGTCCTTCAG; RNA Polymerase F1-CGCTGTGTCTGCTTCTTCTGC, RNA Polymerase R1-TTGCCCTTGCACAGGTCATA; β-Actin F1-ACTGGGACGACATGGAGAAG, β-Actin R1-GACAGCACAGCCTGGATGG. PCR products were run on a 2% agarose gel containing ethidium bromide and visualized using a Gel Doc ™ system (BioRad, Hercules, CA, USA).

### Hematopoietic cell transplantation (HCT)

Mixed breed c*xmd *canines were maintained as a colony at the Fred Hutchinson Cancer Research Center, as previously described [12]. Littermates composed of healthy wild-type donors and c*xmd *recipient dogs were matched by intrafamilial histocompatibility typing, and the HCT protocol for *cxmd *canines was described previously [9]. Briefly, on Day 0 the *cxmd *recipient received 200 cGy TBI at a single dose delivered at 7 cGy/min from a linear accelerator (Varian CLINAC 4, Palo Alto, CA, USA). Within 4 h of TBI, donor bone marrow cells were infused intravenously. On Day 1, freshly isolated donor G-PBMCs were infused intravenously into the *cxmd *recipient. Postgrafting immunosuppression consisted of oral cyclosporine (CSP), 10 mg/kg twice daily, from Days 1 to 35, and 5 mg/kg (MMF) from Days 0 to 7, and 7.5 mg/kg MMF from Days 8 to 28. All dogs were given standard supportive care that included subcutaneous fluids with electrolytes, and systemic antibiotics.

### Canine-to-canine muscle cell transplantation

A muscle biopsy was obtained from the hematopoietic stem cell donor, and muscle-derived cells were isolated as described above for canine-to-murine transplantation experiments. The biceps femoris (BF) muscle of the *cxmd *recipient was marked with non-dissolvable sutures to identify six sites of cell injection. Each suture site was transplanted with donor muscle-derived cells, involving six injections surrounding each suture.

One site from each BF muscle was biopsied 8, 16, and 24 weeks after injection and frozen in OCT (Tissue-Tek, Torrance, CA, USA). Cryosections (8 to 10 μm) were adhered to Superfrost slides (Fisher Scientific), fixed in acetone at -20°C for 10 min, allowed to dry, and rehydrated in PBS. Primary and secondary antibodies used for dystrophin staining, and the method were described above.

## Results

### Canine cells engraft into regenerating mouse muscle

**Figure 1:**
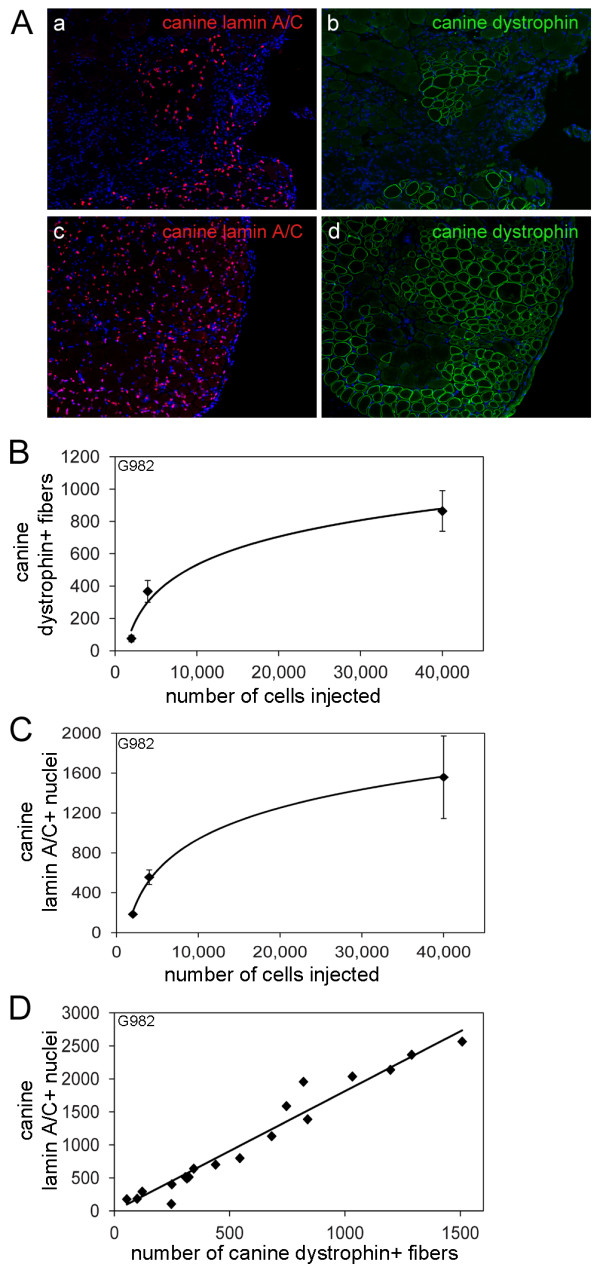
**Canine muscle cell engraftment into mouse muscle is quantifiable and consistent**. **(A) **Cryosections from NOD/SCID mouse muscle injected with 1 × 10^4 ^**(a, b) **or 4 × 10^4 ^**(c, d) **canine muscle-derived mononuclear cells were immunostained with anti-lamin A/C **(a, c)**, or anti-dystrophin (MANDYS107 - **b, d**) and fluorescently labeled secondary antibodies. **(B, C) **Cryosections from mouse muscle injected with canine muscle-derived mononuclear cells were immunostained with anti-lamin A/C or anti-dystrophin (MANDYS107), and fluorescently labeled secondary antibodies. The number of fibers expressing canine dystrophin (B) and the number of nuclei expressing canine lamin A/C (C) per cross-section were counted using cryosections surrounding the region of highest engraftment within the muscle. The points on the graphs represent the average of the averages ± SD, where the average was calculated from three cryosections per mouse, and the average of the averages was calculated from at least three mice per cell dose, and at least two separate cell isolations per cell dose. **(D) **The number of nuclei expressing canine lamin A/C was plotted as a function of the number of fibers expressing canine dystrophin per cross-section.

The lower right hindlimb of each NOD/SCID mouse was exposed to 12 Gy of ionizing radiation, the lowest dose that prevented host muscle regeneration (data not shown). The tibialis anterior (TA) muscle of the same hindlimb was injected with barium chloride to induce muscle regeneration, and the following day, mononuclear cells isolated from a wild-type canine muscle biopsy were injected directly into the same TA muscle, along the length of the muscle, from the proximal to the distal end. The injected muscle was harvested 28 days after injection, and cryosections were immunostained using antibodies specific for canine dystrophin and canine lamin A/C. Muscle injected with 1 × 10^4 ^(Figure 1A a and 1b) or 4 × 10^4 ^(Figure 1A c and 1d) canine cells displayed a significant number of nuclei expressing canine lamin A/C (Figure 1A a and 1c) and fibers expressing canine dystrophin (Figure 1A b and 1d), indicating canine donor cell engraftment.

### Canine donor cell engraftment is quantifiable and consistent

NOD/SCID mice were injected as described above, with freshly isolated canine muscle-derived mononuclear cells from three different donor dogs, with cell doses ranging from 2 × 10^3 ^to 5 × 10^4 ^cells per injection. The number of fibers expressing canine dystrophin and the number of nuclei expressing canine lamin A/C per cross-section of muscle were determined using a minimum of three cross-sections from each injected muscle, from within the region of highest engraftment and covering a distance of approximately 800 to 1200 μm. Muscles from at least two separate experiments were analyzed; each experiment representing a single canine muscle biopsy and cell preparation, and a minimum of three mice for each cell dose.

Initially, we expressed canine donor cell engraftment as a percentage of total fibers expressing canine dystrophin or a percentage of total nuclei expressing canine lamin A/C. However, the amount of host mouse muscle remaining after BaCl_2 _induced degeneration was not consistent between recipients, resulting in variable values when expressing engraftment as a percentage. However, there was a reproducible positive correlation between the number of cells injected from canine donor G982 and the number of fibers expressing canine dystrophin per cross-section (Figure 1B) and the number of nuclei expressing canine lamin A/C per cross-section (Figure 1C). The correlation between cell dose and engraftment was also observed for canine donors G993 and H299 (Additional file 1). A logarithmic curve was determined to be the best fit curve for the data shown in Figures 1B and 1C, and S1 as the r^2 ^value was more than 0.95 for all curves shown. Moreover, a linear relationship between the number of donor nuclei, as determined by expression of canine lamin A/C, and the number of fibers expressing canine dystrophin, was seen for all donors, with an average of approximately 1.75 ± 0.07 canine nuclei per myofiber expressing canine dystrophin per cross-section (Figure 1D, Additional file 1).

Notably, each donor's muscle-derived cell population had a different capacity for reconstitution as measured by the number of fibers expressing canine dystrophin, the number of nuclei expressing canine lamin A/C, and the number of nuclei expressing Pax7 and canine lamin A/C. Yet, different muscle cell preparations from the same donor displayed similar levels of engraftment (Figures 1B and 1C, Additional file 1). Therefore, the canine-to-murine xenotransplantation model provides a sufficiently robust platform to quantitatively assess engraftment potential of different populations of canine muscle cells.

### Canine donor cells consistently engraft to the murine satellite cell niche

**Figure 2:**
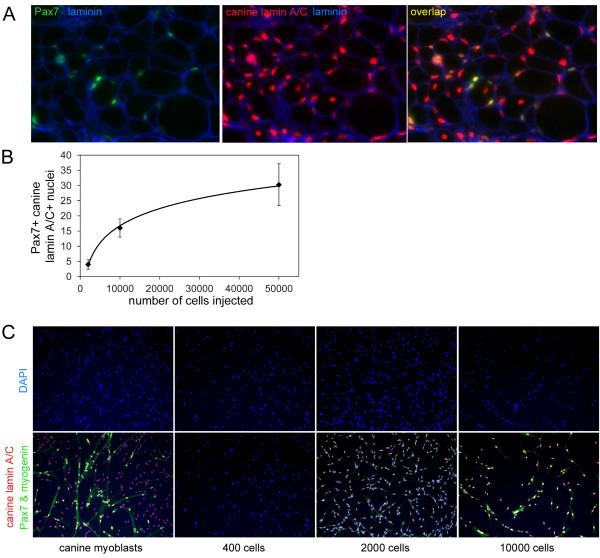
**Canine derived Pax7-positive cells persist in injected mouse muscle**. **(A) **Cryosections from mouse muscle injected with canine muscle-derived mononuclear cells were immunostained with anti-Pax7 (green), anti-lamin A/C (red), and anti-laminin (blue). **(B) **The number of nuclei expressing canine lamin A/C and Pax7 per cross-section was counted, and the points on the graph represent the average of the averages ± SD, where the average was calculated from three cryosections per mouse, and the average of the averages was calculated from at least three mice per cell dose, and at least two separate cell isolations per cell dose. **(C) **Satellite cell-derived myoblasts were isolated and cultured from canine muscle, and mouse muscle injected with 400, 2000, or 10,000 canine muscle-derived mononuclear cells. The cells were fixed and immunostained with anti-lamin A/C (red), and anti-Pax7 and anti-myogenin (both green).

Nuclei expressing Pax7 and canine lamin A/C were detected at the outer periphery of muscle fibers and underneath laminin of the extracellular matrix, suggesting that canine cells had engrafted into the niche normally occupied by murine satellite cells (Figure 2A). In addition, a small number of Pax7-positive nuclei not expressing lamin A/C were detected, indicating that irradiated and injected muscles maintained a minimal population of murine satellite cells (data not shown). Importantly, the number of canine-derived Pax7-positive cells increased with the number of donor cells injected, and engraftment at all cell doses was consistent for each donor (Figure 2B, data not shown).

To confirm that mononuclear cells expressing canine lamin A/C present in transplanted muscle were maintained in the myogenic lineage, mouse TA muscles harvested 28 days after canine muscle cell injection were digested with collagenase, and the resulting mononuclear cells were cultured in growth medium containing 20% fetal bovine serum and 2.5 ng/ml bFGF for 7 to 10 days. Cells were fixed and stained for expression of canine lamin A/C to detect cells of donor canine origin, and for Pax7 and myogenin to detect all myogenic cells. We chose to stain cells for expression of both Pax7 and myogenin to include both undifferentiated and differentiated muscle cells. Cells expressing Pax7 or myogenin isolated and cultured from muscle injected with 2,000 or 10,000 canine donor cells were exclusively canine lamin A/C-positive, indicating that a subpopulation of transplanted canine cells was capable of generating mononuclear muscle cells *in vitro *(Figure 2C). Muscles injected with 400 canine donor cells did not yield detectable numbers of canine muscle cells *in vitro*, perhaps due to the small number of donor cells engrafted.

### A subpopulation of canine muscle-derived cells express CXCR4

**Figure 3:**
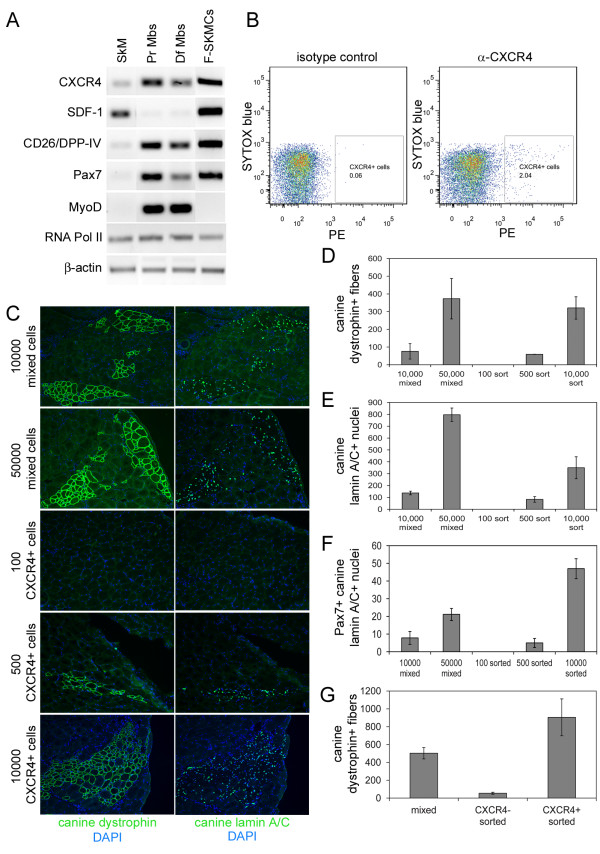
**Sorting for CXCR4-positive cells does not enhance engraftment**. **(A) **RNA isolated from whole canine skeletal muscle (SkM), proliferating canine satellite cell-derived myoblasts (PrMbs), differentiating myoblasts (DfMbs), and freshly isolated canine muscle-derived cells (F-SkMCs), was reverse transcribed and amplified using primers for the genes indicated. **(B) **Canine muscle-derived mononuclear cells were incubated with anti-CXCR4 and PE-labeled secondary antibody, or isotype control and PE-labeled secondary antibody, and SYTOX blue to label dead cells, and sorted using FACS. The population of CXCR4-positive:SYTOX blue-negative cells indicated by the box were collected and prepared for injection. **(C) **Cryosections from mouse muscle injected with 1 × 10^4 ^or 5 × 10^4 ^freshly isolated mixed canine muscle-derived mononuclear cells, or 100, 500, or 1 × 10^4 ^CXCR4-positive sorted cells, were immunostained with anti-dystrophin (MANDYS107) or anti-lamin A/C, and fluorescently labeled secondary antibody. **(D, E, F) **The number of fibers expressing canine dystrophin (D), the number of nuclei expressing canine lamin A/C (E), and the number of nuclei expressing canine lamin A/C and Pax7 (F) per cross-section were determined. The bars represent the average of the averages ± SD, where the average was calculated from three cryosections per mouse, and the average of the averages was calculated from three mice per condition. **(G) **Cryosections from mouse muscle injected with 1 × 10^4 ^freshly isolated mixed canine muscle-derived mononuclear cells, CXCR4-negative sorted cells, or CXCR4-positive sorted cells were immunostained with anti-dystrophin (MANDYS107) and fluorescently labeled secondary antibody. The number of fibers expressing canine dystrophin per cross-section was determined. The bars represent the average of the averages ± SD, where the average was calculated from three cryosections per mouse, and the average of the averages was calculated from three mice per condition.

Recently, a subpopulation of satellite cells expressing CXCR4 have been identified in mice, and cells sorted for expression of CXCR4 and β1-integrin efficiently engraft into regenerating muscle of *mdx *mice [11,13]. We detected expression of CXCR4 transcript in cultured proliferating and differentiating canine satellite cell derived myoblasts, and the freshly isolated population of mixed canine muscle-derived cells (Figure 3A). SDF-1, the sole ligand for CXCR4, was detected in RNA from whole canine skeletal muscle and the freshly isolated population of mixed canine muscle-derived cells, but not cultured myoblasts, suggesting that satellite cells or another population of cells were the source of SDF-1. Notably, Pax7 transcript, but not MyoD transcript, was present in freshly isolated muscle-derived cells, indicating that this population of cells has a more progenitor-like phenotype and has not initiated myogenic commitment and differentiation.

FACS sorting of cultured canine proliferating primary myoblasts demonstrated that 98.5% of viable cells were CXCR4-positive, whereas 88% of viable cells were β1-integrin-positive (data not shown). We sorted freshly isolated canine muscle-derived cells from various donors and observed that approximately 1 to 3.5% of canine muscle-derived mononuclear cells were CXCR4-positive, and less than 0.5% of cells were CD45-positive; however, we were unable to detect a significant population of β1-integrin-positive cells (Figure 3B, data not shown).

### Sorting for CXCR4-positive cells does not increase engraftment efficiency

NOD/SCID mice were injected with freshly isolated mixed canine muscle-derived cells and CXCR4-positive cells sorted from the mixed cell population. Injection of 1 × 10^4 ^CXCR4-positive sorted cells resulted in a greater number of fibers expressing canine dystrophin (Figure 3D), nuclei expressing canine lamin A/C (Figure 3E), and Pax7/canine lamin A/C double-positive nuclei (Figure 3F) per cross-section of muscle when compared to injection of 1x10^4 ^mixed cells. However, for this experiment, 1% of the parent population of mixed cells was CXCR4-positive, and as such, 1 × 10^6 ^mixed cells were required to obtain 1 × 10^4 ^CXCR4-positive sorted cells. Therefore, the true comparison is 1 × 10^4 ^mixed cells to 100 sorted cells, and 5 × 10^4 ^mixed cells to 500 sorted cells. Using this relationship, the freshly isolated mixed canine muscle-derived cells resulted in a greater number of fibers expressing canine dystrophin (Figure 3D), nuclei expressing canine lamin A/C (Figure 3E), and nuclei expressing both Pax7 and canine lamin A/C (Figure 3F).

Recombining CXCR4-positive and CXCR4-negative cells after sorting did not restore engraftment to the level observed with the mixed cell population (data not shown). Moreover, depleting the parent mixed canine muscle-derived cell population of CXCR4-expressing cells also resulted in a 10-fold decrease in the ability to generate fibers expressing canine dystrophin within recipient mouse muscle (Figure 3G), indicating that the CXCR4-positive cells within the mixed cell population are likely responsible for the majority of the observed engraftment, and that sorting may have a negative effect on cell engraftment.

### CXCR4 and SDF-1 play an important role in donor cell engraftment

**Figure 4:**
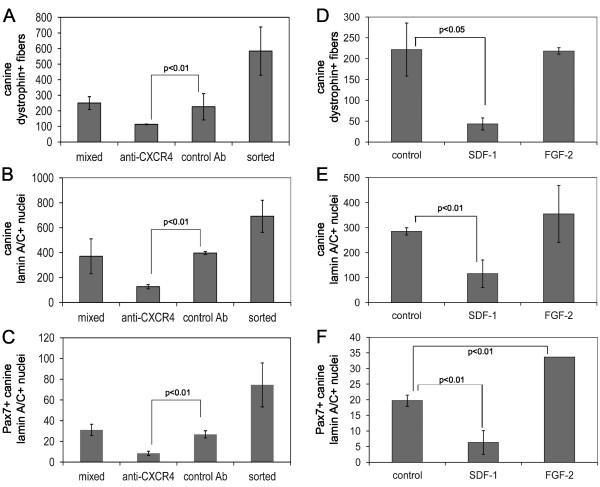
**Anti-CXCR4 antibody and SDF-1 inhibit engraftment**. **(A, B, C) **Cryosections from mouse muscle injected with 1 × 10^4 ^freshly isolated mixed canine muscle-derived mononuclear cells alone, cells pre-incubated with anti-CXCR4, cells pre-incubated with control antibody, or CXCR4-positive sorted cells, were immunostained with anti-dystrophin (MANDYS107) or anti-lamin A/C, and fluorescently labeled secondary antibody. The number of fibers expressing canine dystrophin (A), the number of nuclei expressing canine lamin A/C (B), and the number of nuclei expressing canine lamin A/C and Pax7 (C) per cross-section were determined. The bars represent the average of the averages ± SD, where the average was calculated from three cryosections per mouse, and the average of the averages was calculated from three mice per condition. The *P *values are the result of a Student's t-test. **(D, E, F) **Skeletal muscle cryosections from NOD/SCID mouse muscle injected with 1 × 10^4 ^canine muscle-derived mononuclear cells alone, or cells incubated with 10 ng/ml SDF-1 or FGF-2 before injection, were immunostained with anti-dystrophin (MANDYS107) or anti-lamin A/C, and fluorescently labeled secondary antibody. The number of fibers expressing canine dystrophin (D), the number of nuclei expressing canine lamin A/C (E), and the number of nuclei expressing canine lamin A/C and Pax7 (F) per cross-section were determined. The bars represent the average of the averages ± SD, where the average was calculated from three cryosections per mouse, and the average of the averages was calculated from three mice per condition. The *P *values are the result of a Student's t-test.

The process of sorting cells may adversely affect engraftment by reducing cell viability; however, it is also possible that binding of the antibody to the cell surface CXCR4 receptor interferes with function. Muscle injected with freshly isolated mixed canine muscle-derived cells incubated with α-CXCR4 antibody, but not subjected to FACS, displayed significantly fewer myofibers expressing canine dystrophin (Figure 4A), fewer nuclei expressing canine lamin A/C (Figure 4B), and fewer nuclei expressing Pax7 and canine lamin A/C (Figure 4C) than muscle injected with cells alone or cells incubated with control antibody. This may be due to antibody-mediated internalization of the receptor or blocking of SDF-1 binding to the CXCR4 receptor [14-17]. Either mechanism argues for an important role for CXCR4 in engraftment and/or differentiation of donor cells.

The function of the CXCR4 receptor requires binding of its ligand, SDF-1. We compared engraftment of the freshly isolated mixed canine muscle-derived cells alone to mixed cells incubated before injection with 10 ng/ml of SDF-1 or 10 ng/ml of FGF-2. SDF-1, but not FGF-2, specifically reduced the number of fibers expressing canine dystrophin (Figure 4D), the number of nuclei expressing canine lamin A/C (Figure 4E), and the number of nuclei expressing Pax7 and canine lamin A/C (Figure 4F). Therefore, exogenously added SDF-1 did not improve donor cell engraftment, and indeed, appeared to mimic the effect of the anti-CXCR4 antibody. It is intriguing to hypothesize that exogenous SDF-1 blocked binding of CXCR4 on canine donor cells to SDF-1 in recipient mouse muscle.

### Diprotin A treatment of donor cells enhances engraftment through CXCR4

Binding of SDF-1 to CXCR4 is negatively regulated by CD26/DPP-IV, a cell surface peptidase that cleaves SDF-1 at the N-terminus [18,19]. CD26 is expressed on hematopoietic stem cells, and inhibition of peptidase activity with diprotin A enhances engraftment of donor hematopoietic cells to the recipient bone marrow niche, presumably by strengthening the interaction between CXCR4 on the surface of donor stem cells and SDF-1 present in the bone marrow niche [20,21].

**Figure 5:**
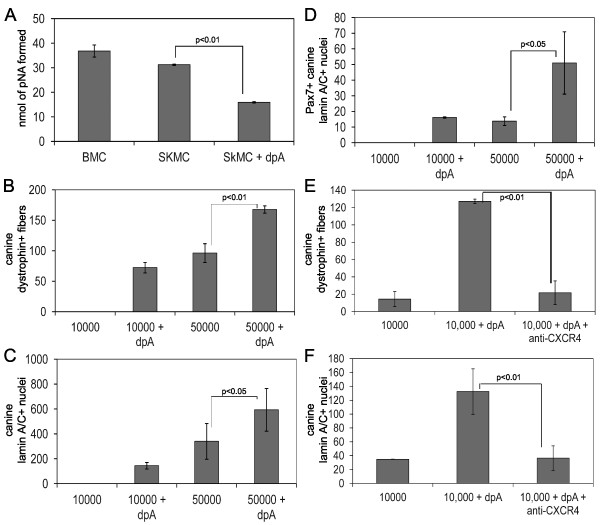
**Diprotin A enhances engraftment of donor cells**. **(A) **1 × 10^5 ^canine bone marrow cells (BMC), and 1 × 10^5 ^freshly isolated skeletal muscle-derived cells (SkMC) alone, or with 5 mM diprotin A (SkMC+dpA), were incubated with Gly-Pro-p-nitroaniline for 2 h. The nmol of p-nitroaniline (pNA) produced by peptidase cleavage was determined by measuring absorbance at 405 nm and converting the value to nmol of pNA using a standard curve. The bars represent the average nmol of pNA produced from three separate samples, and the *P *values are the result of a Student's t-test. **(B, C, D) **Cryosections from mouse muscle injected with 1 × 10^4 ^or 5 × 10^4 ^canine muscle-derived mononuclear cells alone or cells treated with 5 mM diprotin A were probed with anti-dystrophin (MANDYS107), anti-lamin A/C, or anti-lamin A/C and anti-Pax7, and fluorescently labeled secondary antibody. The number of fibers expressing canine dystrophin (B), the number of nuclei expressing canine lamin A/C (C), and the number of nuclei expressing canine lamin A/C and Pax7 (D) per cross-section were determined. The bars represent the average of the averages ± SD, where the average was calculated from three cryosections per mouse, and the average of the averages was calculated from three mice per condition. The *P *values are the result of a Student's t-test. **(E, F) **Cryosections from mouse muscle injected with 1 × 10^4 ^freshly isolated mixed canine muscle-derived mononuclear cells alone, cells treated with 5 mM diprotin A, or cells treated with 5 mM diprotin A and α-CXCR4 antibody, were probed with anti-dystrophin (MANDYS107), or anti-lamin A/C, and fluorescently labeled secondary antibody. The number of fibers expressing canine dystrophin (E) and the number of nuclei expressing canine lamin A/C (F) per cross-section were determined. The bars represent the average of the averages ± SD, where the average was calculated from three cryosections per mouse, and the average of the averages was calculated from three mice per condition. The *P *values are the result of a Student's t-test.

RT-PCR demonstrated that cultured proliferating and differentiating canine satellite cell derived myoblasts, and the freshly isolated population of mixed muscle-derived cells expressed CD26/DPP-IV transcript (Figure 3A). Freshly isolated canine bone marrow cells, a positive control for CD26/DPP-IV activity, and muscle-derived cells were incubated with Gly-Pro-p-nitroanilide, a substrate of CD26/DPP-IV, and production of the cleavage product, p-nitroaniline, was monitored by measuring absorbance at 405 nm. Muscle-derived mononuclear cells displayed CD26/DPP-IV activity at a level comparable to canine bone marrow cells, and this activity was inhibited with diprotin A (Figure 5A).

Diprotin A treatment of donor cells before injection significantly increased the number of fibers expressing canine dystrophin (Figure 5B), the number of nuclei expressing canine lamin A/C (Figure 5C), and the number of Pax7/canine lamin A/C double-positive nuclei detected in injected muscle (Figure 5D). To confirm the increase in donor nuclei present, a PCR-based assay that distinguishes between canine and mouse DNA showed that mouse muscle injected with diprotin A treated donor canine cells had three-fold more canine DNA content than muscle injected with cells alone (data not shown).

However, CD26/DPP-IV cleaves other chemokines, such as IP-10/CXCL10 and MIP1β/CCL4 [22,23]. To ensure that the increase in engraftment we observed with diprotin A was specific for SDF-1/CXCR4, canine muscle-derived cells were incubated with diprotin A alone or with diprotin A and α-CXCR4 antibody before injection. As shown in Figures 5E and 5F, the increase in engraftment in the presence of diprotin A was prevented by α-CXCR4 antibody, indicating that diprotin A specifically affected binding of SDF-1 to CXCR4.

### Diprotin A increases engraftment of donor derived Pax7-positive cells

**Figure 6:**
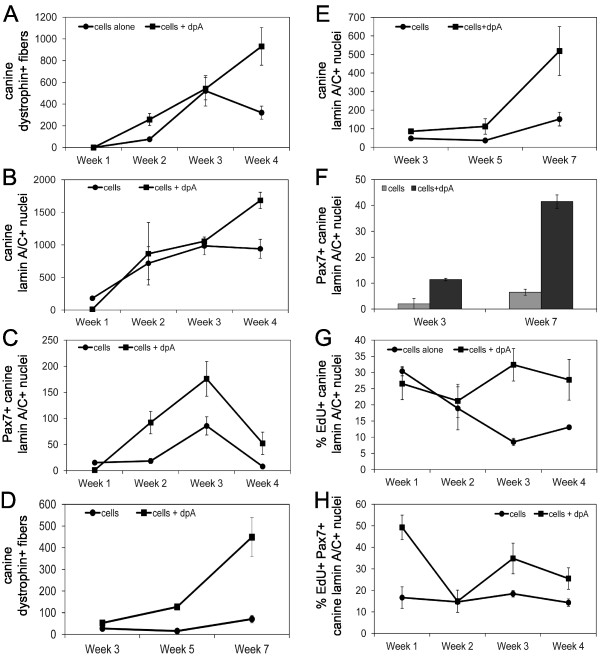
**Diprotin A enhances proliferation of donor cells *in vivo***. Mouse muscle injected with 1 × 10^4 ^canine muscle-derived mononuclear cells alone, or cells treated with 5 mM diprotin A, were harvested weekly for 4 weeks after injection **(A, B, C)**, or at 3, 5, or 7 weeks after injection **(D, E, F) **and cryosections probed with anti-dystrophin (MANDYS107), anti-lamin A/C, and/or Pax7, and fluorescently labeled secondary antibody. The number of fibers expressing canine dystrophin (A, D), and the number of nuclei expressing canine lamin A/C (B, E), and the number of nuclei co-expressing Pax7 and canine lamin A/C (C, F) per cross-section were determined, and the points represent the average of the averages ± SD, where the average was calculated from three cryosections per mouse, and the average of the averages was calculated from three mice per condition. **(G, H) **Mice injected with 1 × 10^4 ^canine muscle-derived mononuclear cells alone, or cells treated with 5 mM diprotin A, were given EdU daily for 1 week during weeks 1, 2, 3, or 4 after injection. Injected muscle was harvested after the last dose of EdU, and cryosections stained for canine lamin A/C, or Pax7 and canine lamin A/C, and EdU. The percentage of canine lamin A/C-positive nuclei that incorporated EdU (G) and the percentage of canine lamin A/C:Pax7 double positive nuclei that incorporated EdU (H) per cross-section were determined. The points represent the average of the averages ± SD, where the average was calculated from three cryosections per mouse, and the average of the averages was calculated from three mice per condition. The *P *value is the result of a Student's t-test.

NOD/SCID mice were injected as described above with 1 × 10^4 ^untreated freshly isolated mixed canine muscle-derived cells, or 1 × 10^4 ^diprotin A treated cells, and engraftment was measured weekly for 4 weeks (Figure 6A, B, C), or at weeks 3, 5, and 7 after injection (Figure 6D, E, F). Different donors were used for the experiment shown in Figure 6A, B, Cand the experiment shown in Figure 6D, E, F, which resulted in a different level of engraftment. However, the trend in engraftment as a function of time, and the effect of diprotin A is similar.

In muscle injected with untreated cells, the number of canine dystrophin-positive fibers reached a plateau 3 weeks after injection, and remained constant from weeks 3 through 7 (Figure 6A and 6D). In contrast, muscle injected with diprotin A-treated cells displayed a continuous increase in canine dystrophin-positive fibers for no less than 7 weeks after donor cell injection, and beyond week 3, displayed a significantly greater number of canine dystrophin-positive fibers compared to muscle injected with untreated cells (Figure 6A and 6D).

A similar pattern is observed for the number of nuclei expressing canine lamin A/C per cross-section (Figure 6B and 6E). However, the number of Pax7/canine lamin A/C double-positive cells are significantly increased in muscle injected with diprotin A-treated cells at all time points (Figure 6C and 6F). Moreover, there is a significant increase in the number of Pax7/canine lamin A/C double-positive cells between weeks 1 and 2 in muscle injected with diprotin A-treated cells, suggesting that Pax7/canine lamin A/C double-positive cells proliferated *in vivo*.

### Diprotin A maintains donor cell proliferation

To quantitatively assess proliferation of donor cells *in vivo*, the nucleoside analog, EdU, was administered daily for 7 days, during weeks 1, 2, 3, or 4 after injection of untreated cells or diprotin A-treated cells into NOD/SCID mice. During weeks 1 and 2, both EdU-positive and EdU/canine lamin A/C double-positive nuclei were observed; however, EdU-positive cells that did not co-express canine lamin A/C were rare in weeks 3 and 4, suggesting that proliferating cells present during weeks 3 and 4 were mainly donor-derived (data nor shown).

The proportion of EdU/canine lamin A/C double-positive cells decreased in muscle injected with untreated cells and reached a plateau of approximately 10% by week 3 (Figure 6G). In contrast, diprotin A treatment of donor cells resulted in an increase in the proportion of donor cells incorporating EdU at weeks 3 and 4 after injection, reaching approximately 25 to 30% of cells. Less than 1% of cells co-expressed canine lamin A/C and caspase-3 at weeks 1 and 4 after injection, and diprotin A treatment did not affect the number of canine donor cells expressing caspase-3 (data not shown). This is consistent with the view that the majority of donor cell death occurs in the first 24 h after injection [24,25]. Together, these data indicate that diprotin A treatment maintained a greater proportion of donor cells in proliferation.

The proportion of EdU/canine lamin A/C double-positive cells that also express Pax7 remained constant at approximately 16% in muscle injected with untreated cells (Figure 6H). In muscle injected with diprotin A-treated cells, this proportion increased to 35% at week 3, and remained significantly higher than in muscle injected with untreated cells. This indicates that diprotin A treatment maintains a greater proportion of proliferating donor derived Pax7-positive myogenic cells *in vivo*.

### Diprotin A enhances engraftment of donor cells in immune tolerant *cxmd *canines

Together, these current data indicate that the most effective regimen involves intramuscular transplantation of a freshly isolated mixed population of canine muscle-derived cells treated with diprotin A before injection. Therefore, to determine if these results can be translated to a large animal model of muscular dystrophy, we chose to compare engraftment of freshly isolated mixed muscle-derived cells alone to cells treated with diprotin A using the immune tolerant *cxmd *canine model of DMD.

Two *cxmd *canines underwent hematopoietic stem cell transplantation (HSCT), as previously described [9]. Briefly, each *cxmd *recipient was exposed to 200 cGy of total body irradiation, and infused with bone marrow and G-CSF mobilized peripheral blood mononuclear cells from a DLA-identical littermate donor. For the first *cxmd *recipient, H376, analysis of donor chimerism 4 weeks after transplant demonstrated that 80% of granulocytes and 46% of lymphocytes were donor derived. However, 12 weeks after transplant, donor hematopoietic chimerism increased to 99% for granulocytes and 80% for lymphocytes and remained constant thereafter. The donor muscle cell injection was performed 15 weeks after bone marrow transplantation. A muscle biopsy was obtained from the hematopoietic stem cell donor, and muscle-derived cells were isolated as described above for canine-to-murine transplantation experiments.

The biceps femoris (BF) muscle on one side of the *cxmd *recipient was marked with non-dissolvable sutures to identify six sites of cell injection. In an attempt to mimic the xenotransplant regimen, three sites were injected with 5 × 10^5 ^cells resuspended in 1.2% barium chloride, and three sites with 5 × 10^5 ^diprotin A-treated cells resuspended in 1.2% barium chloride. The sites injected with untreated cells were separated from the sites injected with diprotin A-treated cells by a minimum of 5 cm to prevent crossover or contamination.

**Figure 7:**
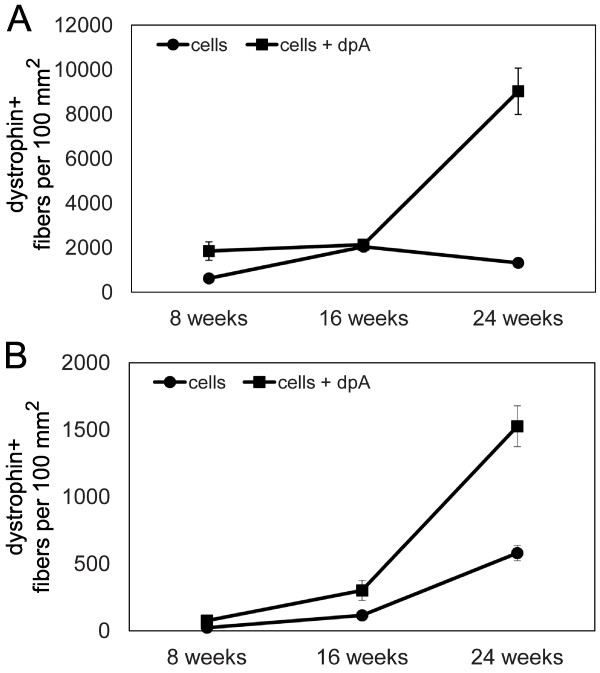
**Diprotin A enhances engraftment of donor cells in canine allogeneic transplantation**. **(A) **Skeletal muscle cryosections from a chimeric *cxmd *canine (H376) biopsied 8, 16, and 24 weeks after injection with 5 × 10^5 ^donor cells in 1.2% BaCl_2_, or 5 × 10^5 ^donor cells treated with diprotin A in 1.2% BaCl_2 _were immunostained with anti-dystrophin and fluorescently labeled secondary antibodies. The number of fibers expressing canine dystrophin was determined and the points represent the average number of fibers expressing dystrophin ± SD (*n *= 5 cryosections from each biopsy). **(B) **Skeletal muscle cryosections from a chimeric *cxmd *canine (H220) biopsied 8, 16, and 24 weeks after injection with 4 × 10^5 ^donor cells or 4 × 10^5 ^donor cells treated with diprotin A were immunostained with anti-dystrophin and fluorescently labeled secondary antibodies. The number of fibers expressing canine dystrophin was determined and the points represent the average number of fibers expressing dystrophin ± SD (*n *= 5 cryosections from each biopsy).

Injected muscle was biopsied 8, 16, and 24 weeks after injection. The average number of dystrophin-expressing fibers per cross-section of muscle was determined using five cryosections from each biopsy, covering a total distance of 1000 μm, and normalized to cross-sectional area. Diprotin A treatment of donor cells resulted in a dramatic 6.8-fold increase in the number of fibers expressing dystrophin 24 weeks after injection, recapitulating the effect observed in canine-to-murine xenotransplantation experiments (Figure 7A).

To determine if the active muscle regeneration induced by barium chloride was important for donor cell engraftment, a second chimeric *cxmd *recipient, H220, was injected with cells resuspended in PBS alone. Analysis of donor chimerism in H220 8 weeks after HSCT demonstrated that 51% of granulocytes and 16% of lymphocytes were donor derived. However, 12 weeks after transplant, donor hematopoietic chimerism dropped to 3% and remained constant thereafter. The donor muscle cell injection was performed 37 weeks after HSCT. The biceps femoris (BF) muscles on each side of the *cxmd *recipient were marked with non-dissolvable sutures to identify six sites of cell injection. The left BF muscle was injected with 4 × 10^5 ^cells alone, and the right BF muscle injected with 4 × 10^5 ^diprotin A-treated cells.

Biopsies of injected muscle were obtained 8, 16, and 24 weeks after injection. Diprotin A treatment of donor cells resulted in a 2.6-fold increase in the number of dystrophin-positive fibers 24 weeks after injection (Figure 7B). The number of dystrophin-positive fibers 24 weeks after injection was 15.3 per mm^2 ^for H220 as compared to 90.3 per mm^2 ^for H376, suggesting that co-injection of cells with barium chloride enhanced engraftment of donor cells. Together, these data indicate that diprotin A successfully enhanced engraftment of donor cells to dystrophic skeletal muscle, and that results from the xenotransplant model accurately predicted results from the canine-to-canine allogeneic model.

## Discussion

Transplantation of myogenic stem cells possesses great potential for long-term repair of dystrophic muscle. We previously established an immune tolerant canine model of muscular dystrophy using *cxmd *canines to test the feasibility of allogeneic muscle cell transplantation, and demonstrated that intramuscular injection of donor cells restored expression of dystrophin for at least 24 weeks in the absence of post-transplant immunosuppression. This is an important pre-clinical model as the phenotype of the canine model of DMD (*cxmd*) faithfully recapitulates the human disease, and is expected to respond to therapeutic intervention in a way that is similar to human dystrophic muscle. Indeed, these dogs have been successfully used to test viral-mediated gene delivery and anti-sense oligonucleotide induced exon skipping approaches for restoring dystrophin function prior to human clinical trials [26-31]. Specifically, studies of gene therapy in *cxmd *canines revealed an unexpected immune reaction not observed in mice, which appears to be recapitulated in DMD patients [32].

However, the canine-to-murine xenotransplant model is a more rapid and cost-effective means of quantitatively comparing donor cell engraftment. Importantly, we show that the canine-to-murine xenotransplant model accurately predicted results of allogeneic transplantation involving the *cxmd *canine model of muscular dystrophy. Once we identify the most effective cell population, canine-to-canine allogeneic transplantation will allow us to address the problems of limited spread of donor cells from the sites of injection, as well as dysregulation of signalling pathways that affect muscle regeneration. Therefore, the xenotransplant model will prioritize the most effective regimens and facilitate translation to the pre-clinical immune tolerant *cxmd *canine model of DMD.

These data also suggest that a human-to-murine xenotransplant model will accurately predict results of donor muscle cell transplantation in clinical trials. A number of groups have successfully established human-to-murine xenotransplant models, mainly using cultured human myoblasts [33-37]. We will also generate a human-to-murine xenotransplant model using freshly isolated muscle cells, and translate the most effective canine regimens to this model to confirm that equivalent human cell populations engraft and respond as predicted by the canine studies.

We used the canine-to-murine xenotransplant model to clearly demonstrate that CXCR4 plays an important role in canine donor cell engraftment, confirming results observed in murine-to-murine transplantation studies [10,11]. CXCR4 binds to its ligand, SDF-1, dimerizes, and activates downstream kinases, including focal adhesion kinase (FAK), which has been shown to regulate expression of caveolin-3 and β1-integrin, genes essential for myoblast fusion [38-44]. Indeed, *in vitro *studies using primary mouse myoblasts demonstrate that CXCR4/SDF-1 is required for proper myogenic fusion, yet also regulates migration of both proliferating and terminally differentiated muscle cells [45].

A role for CXCR4 in migration of muscle cells suggests that CXCR4 binding to SDF-1 is involved in homing of muscle cells to a niche. Studies in murine and avian systems have clearly shown that myogenic progenitor cells express CXCR4 and migrate towards regions of SDF-1 expression during embryonic limb muscle development [46,47]. Expression of SDF-1 and heparin sulfate proteoglycans (HSPGs) increases during the first 3 to 5 days after induction of muscle regeneration, presumably functioning to attract satellite cells and/or immune cells to the site of damage, both of which are required for regeneration [45,48,49]. We clearly show that diprotin A treatment of donor cells resulted in an increased number of Pax7/canine lamin A/C double-positive cells within injected muscle, suggesting that diprotin A stimulated engraftment of donor cells to a "niche" within regenerating muscle. This is consistent with studies demonstrating that diprotin A stimulates homing and adhesion of donor hematopoietic stem cells (HSCs) to the bone marrow niche [21,50].

However, expression of Pax7 is not confined to quiescent satellite cells, and as such the number of Pax7/canine lamin A/C double-positive cells may not accurately measure the number of donor cells residing in the host satellite cell niche, but rather measures the number of donor derived myogenic mononuclear cells, either quiescent or proliferating. Although this does not contradict the hypothesis that diprotin A enhances donor cell engraftment to a niche, it does suggest that diprotin A may have improved donor cell survival after injection, or induced donor cell proliferation *in vivo*. Indeed, the proportion of Pax7/canine lamin A/C double-positive cells that incorporate EdU is significantly higher in muscle injected with diprotin A-treated cells, indicating a role for diprotin A in stimulating donor cell proliferation.

Currently, a clinical trial is underway at Indiana University School of Medicine using sitagliptin, a CD26/DPP-IV inhibitor, to enhance engraftment of cord blood stem cells in HSC transplant for patients with hematological malignacies. Sitagliptin is FDA-approved for use in patients to lower blood sugar levels, and as a DPP-IV inhibitor, may also be able to enhance engraftment of donor cells to muscle of patients with muscular dystrophy. We will use the canine-to-murine and human-to-murine xenotransplantation model, as well as the canine-to-canine allogeneic transplant model, to test the efficacy of this inhibitor in preclinical studies.

## Conclusions

The canine-to-murine xenotransplantation model accurately predicted the increase in donor cell engraftment observed in allogeneic canine-to-canine transplantation after treatment of donor cells with diprotin A to inhibit CD26/DPP-IV peptidase activity. Importantly, diprotin A treatment stimulated sustained donor cell proliferation *in vivo*, resulting in an increased number of fibers expressing dystrophin, and the potential for long-term therapeutic benefit. Furthermore, our results suggest the potential pre-clinical utility of a human-to-mouse xenotransplantation model in assessing the relative efficacy of human muscle stem cell populations.

## Abbreviations

DMD: Duchenne muscular dystrophy; PBS: phosphate buffered saline; HSCT: hematopoietic stem cell transplant; DPP-IV: dipeptidylpeptidase IV; SDF-1: stromal derived factor 1; NOD/SCID: non-obese diabetic/severe combined immunodeficiency; TA: tibialis anterior; BF: biceps femoris; FACS: fluorescence activated cell sorting.

## Competing interests

The authors declare that they have no competing interests.

## Authors' contributions

MHP participated in the conception and design of the study, execution of the experiments, and preparation of the manuscript. CL, AT, and LS assisted in execution of the experiments. RS participated in conception and design of the study. SJT participated in conception and design of the study, and preparation of the manuscript. All authors read and approved the final manuscript.

## Supplementary Material

Additional file 1 **Canine muscle cell engraftment into mouse muscle is quantifiable and consistent**. This file shows specificity of the dystrophin and lamin A/C antibodies used, and provides quantitative engraftment data for muscle-derived cells from additional donor canines. Click here for file
