# Supplementary material for: Inhibition of CD26/DPP-IV enhances donor muscle cell engraftment and stimulates sustained donor cell proliferation
Source: Skelet Muscle. 2012 Feb 16;2:4. doi: 10.1186/2044-5040-2-4 (PMC3299591; doi:10.1186/2044-5040-2-4)
Supplement: Additional file 1 — Canine muscle cell engraftment into mouse muscle is quantifiable and consistent. This file shows specificity of the dystrophin and lamin A/C antibodies used, and provides quantitative engraftment data for muscle-derived cells from additional donor canines. [file 2044-5040-2-4-S1.DOC]

# Inhibition of CD26/DPP-IV Enhances Donor Muscle Cell Engraftment and Stimulates Sustained Donor Cell Proliferation

**Running title:** CD26 inhibition enhances muscle cell engraftment

Maura H. Parker^1^*, Carol Loretz^1^, Ashlee Tyler^2^, Lauren Snider^2^, Rainer Storb^1,3^, and Stephen J. Tapscott^2,4^

Additional file 1.


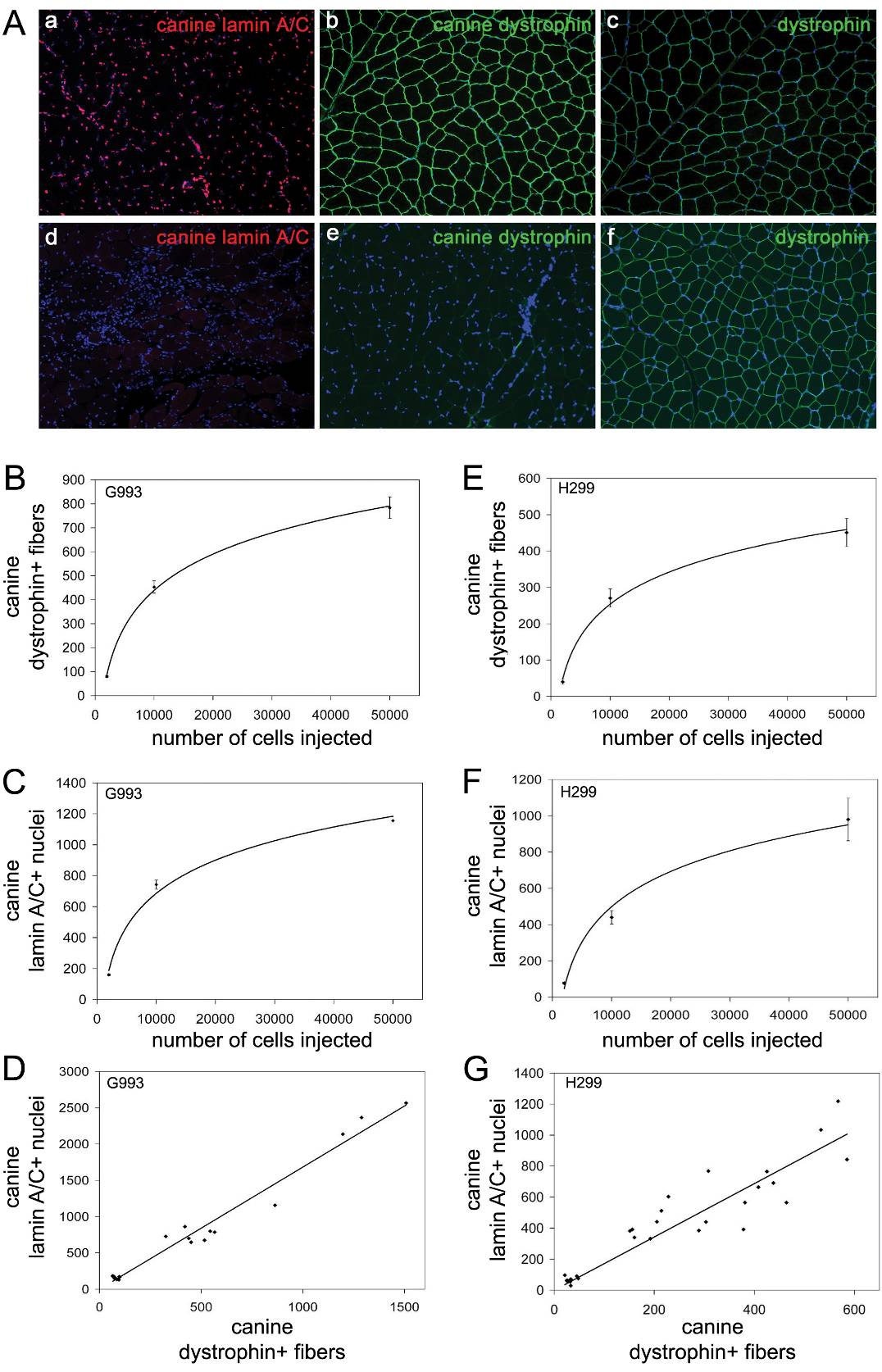


(**A**) Canine (a,b,c) and mouse (d,e,f) skeletal muscle cryosections were immunostained with anti-lamin A/C (a,d), or anti-dystrophin (MANDYS107 – b,e, MANEX1A – c,f) and fluorescently labeled secondary antibodies. Anti-lamin A/C and anti-dystrophin (MANDYS107) recognize canine but not mouse antigens. (**B-G**) Skeletal muscle cryosections from NOD/SCID mouse muscle injected with muscle derived mononuclear cells from canine donor G993 (**B-D**) or H299 (**E-G**), were immunostained with anti-lamin A/C, or anti-dystrophin (MANDYS107), and fluorescently labeled secondary antibodies. The number of fibers expressing canine dystrophin (**B,E**) and the number of nuclei expressing canine lamin A/C (**C,F**) were counted using cryosections surrounding the region of highest engraftment within the muscle. The points represent the average of the averages ± SD, where the average was calculated from 3 cryosections per mouse, and the average of the averages was calculated from at least 3 mice per condition, and at least 2 separate cell isolations per cell dose. The p-value is the result of a Student’s t-test. (**D,G**) The number of nuclei expressing canine lamin A/C was plotted as a function of the number of fibers expressing canine dystrophin per cross-section for G993 (**D**) and H299 (**G**). An average of 1.75±0.7 canine lamin A/C nuclei is present per canine dystrophin-positive muscle fiber for all donors.
